# Epidemic Cholera in Indiana

**Published:** 1834

**Authors:** W. T. S. Cornett


OF THE

MEDICAL AND PHYSICAL SCIENCES.

APRIL, MAY, AND JUNE, 1834.

### ESSAYS AND CASES.

### Art. I.—Epidemic Cholera in Indiana.

*Brief Remarks on Epidemic Cholera as it appeared in Versailles, Indiana, and its vicinity, in the summer of* 1833. By W. T. S. Cornett, M. D.

In order that the treatment of this terrible scourge of the human family may become certain and settled, it is necessary that those of the profession who have seen, and treated the disease, should give to the public the result of their observations, even at the expense of being tedious. We have all read of Cholera until we are weary of it. The bare caption of an article on that topic, is almost sufficient to bring on a fit of ennui, but the advancement of science, the mitigation of human suffering, and the credit of the profession, all demand patient investigation at our hands. The accounts of the *methodus medendi* in this disease are still, as heretofore, various and conflicting, and calculated to leave in confusion the minds of those who have never as yet seen and treated the disease for themselves. Under these considerations, I am induced to lay before the profession a very brief account of the treatment practised by myself, with its results. The epidemic which commenced here in June last, was preceded for several weeks by bowel complaints, which yielded readily to a few doses of calomel and opium, confinement to bed, fomentations to the abdomen, &c. During the prevalence of the epidemic the digestive organs of nearly all the inhabitants were more or less affected. Those who were not afflicted with Diarrhœa, complained of uneasiness at the stomach, such as is experienced from indigestion, and occasional pain in the bowels. After eating, the aliment would remain for a time in the stomach, undergoing but slightly the process of digestion, and then be expelled through the pylorus with a gurgling noise, leaving a sense of vacuity in the stomach, and a disagreeable sense of sinking in of the præcordia. This feeling in many was accompanied by instinctive desire for something stimulating, ei[illegible]r in the way of drink or condiment. I frequently observed when standing in a crowd of men in the streets, borborigmi in the bowels of the whole company, so audible as to attract general attention. An apparent purging would commence frequently, in the upper tract of the bowels, so as to induce the belief that diarrhœa was commencing, but this action extended in many instances, no farther than the ileo-colic valve. A number with whom I conversed, complained of double vision. This phenomenon occurred indiscriminately, with those laboring under the disease, and also with persons who experienced no attack.

The crude vegetables of the season seemed to be inappropriate articles of diet, the stomach being entirely incapable of digesting them. I have seen green apples, snap beans, &c. vomited up, having remained four or five days in the stomach without alteration in appearance.

The disease was in most cases preceded by diarrhœa, but not uniformly. Some of the worst attacks were sudden as an electric shock, without any premonitory symptoms whatever, save indigestion, and a sense of purging in the upper tract of the bowels. Having made these preliminary observations I shall proceed at once to a summary of the treatment which proved most successful in my hands; and in short, I may say, that from an extensive practical experience in this disease, calomel in large doses is the only remedy, which has proved succesful in my hands. I was led at the onset of this disease, to an apparently extravagant use of this article, from the entire suppression of the biliary and other secretions. At first, I used opium in conjunction with calomel, with the view of restraining the watery evacuations from the intestines, but soon ascertained that calomel alone was sufficient, when given in proper doses, not only to restore ultimately the lost secretions, but at once to arrest the diarrhœa, and save from the impending collapse. I therefore abandoned opium as useless, and saw some cases in which it was manifestly injurious. I commenced the treatment in most cases with an emetic, with a view of clearing the stomach of all crudities; for which purpose I used common salt, and mustard, or the latter alone. This emetic always acted promptly, and possessed the advantage of not producing that debilitating nausea common to the antimonial preparations. I then commenced giving calomel, in doses of from 50 to an 100 grains, every hour or two, until diarrhœa was arrested; then the dose was reduced to 25 grains every two hours, until ptyalism was effected, which was always followed by a restoration of the secretions in general, when the patient was out of danger. I will here remark, that out of largely upwards of an hundred cases, I lost but one patient who had sore mouth, and that was dry inflammation of the gums, perhaps a local affection, as the breath was destitute of the mercurial fætor. I made it a rule in nearly all cases, to push the mercurial course until salivation was brought about, after which, I saw no death, no relapse, nor any case of intractable sore mouth. Indeed, I can safely say, that salivations produced with those large doses of calomel, are more mild, and sooner cured, than those effected with smaller quantities. After having arrested diarrhœa, and reduced the calomel to 25 grains every two hours, if it returned, I immediately increased the quantity, until the intestinal effusion was again arrested. There is a constant tendency to a return of this symptom until the secretions are restored, and it should be strictly watched, and immediately counteracted when it occurs. Upon this depends the success of the treatment. The intestinal effusion is the chief cause of the collapse, and when this is held in check, there is no fear of immediate death. I used blood-letting and blistering in some cases, but with no marked good effect. When spasms were present, I found advantage from large sinapisms to the extremities and epigastrium. The mustard emetic was the most valuable remedy for cramp, acting in most cases like a charm, driving the circulation to the surface, and completely throwing off the spasms. I used many auxiliary means in the earlier part of the epidemic, but soon abandoned them as useless. Calomel—and calomel alone, is the great *sine qua non*, in the treatment of this disease; and will, when properly administered, cure 49 out of 50 cases, without auxiliary treatment. This remark is not carelessly made, but with entire confidence in its truth, derived from careful observation at the bed side. Out of largely upwards of an hundred cases, I lost ten, eight of whom were not seen until a late date. Least it should be judged that the disease here was unusually mild, I will remark, that at the Cross Plains, a village nine miles south of Versailles, every case terminated fatally, until the above practice was adopted at my instance; after which, not a case was lost, where the treatment was early resorted to. For the truth of these remarks I can confidently appeal to my fellow citizens, many of whom, instead of being afflicted with that panic which prevailed so generally in many places, labored day and night in nursing, and also in prescribing with equal success, when medical aid was not to be had.

To recapitulate. Calomel is to be given in such doses as will arrest the diarrhœa, without regard to quantity: the doses may then be diminished, but should be continued until ptyalism is effected, after which, there is no danger of relapse. This should always be brought about. Who would not prefer a sore mouth and safety, to the risk of a relapse and its consequences? I have seldom seen the secretions properly established until the mercurial impression was made. There is no telling, (*a priori*,) the quantity of calomel that will be required. In some cases I have been under the necessity of giving the fourth of a pound;* whilst in others, a much less quantity was sufficient. It is given to answer certain ends, and those ends must be answered, take what quantity it may. Where children were afflicted with the disease, I found it more difficult to make the mercurial impression. In those cases, frictions along the spine, with the Ungt. Hydrarg. will be found a valuable auxiliary.

*May*, 1834.

*Calomel has no tendency to act as a purge in this disease until the secretion of bile is restored.

